# Breaking the compatibility barrier: cholangioscopy-guided laser lithotripsy during enteroscopy-assisted endoscopic retrograde cholangiopancreatography in Roux-en-Y anatomy

**DOI:** 10.1055/a-2965-5835

**Published:** 2026-10-07

**Authors:** Alda Andrade, Ana Rita Ribeiro, Jorge Bezerra, Marta Moreira, Jorge Canena, Tarcísio Araújo, Luís Lopes

**Affiliations:** 1 Gastroenterology Department, Hospital Santa Luzia 208305 ULS Alto MinhoViana do CasteloPortugal; 2 Gastroenterology Department 679534 Unidade Local de Saúde de Amadora/SintraAmadoraPortugal; 3 599930 Hospital CUF TejoLisbonPortugal; 4 ICVS/3B's - PT Government Associate Laboratory 511313 Braga/GuimarãesPortugal; 5 Life and Health Sciences Research Institute (ICVS) 224744 School of Medicine, University of MinhoBragaPortugal

Double-balloon enteroscopy–assisted endoscopic retrograde cholangiopancreatography (DBE-ERCP) has significantly improved biliary access in patients with surgically altered anatomy. However, cholangioscopy-guided therapy has remained technically challenging because the diameter and working length of conventional cholangioscopes have historically precluded their use through short double-balloon enteroscopes. The recent availability of slim digital cholangioscopy systems has enabled advanced intraductal therapy during DBE-ERCP. 1​

A 67-year-old man with a history of subtotal gastrectomy with Roux-en-Y reconstruction for gastric adenocarcinoma presented with persistent cholestatic liver enzyme elevation. Computed tomography demonstrated a 15-mm dilated common bile duct with intrahepatic biliary dilatation secondary to two 10-mm bile duct stones. Initial DBE-ERCP confirmed two large filling defects; however, stone extraction using conventional techniques was unsuccessful. A plastic biliary stent was therefore placed to ensure biliary drainage, and a second procedure was scheduled. At repeat DBE-ERCP 2 months later, a slim digital cholangioscope (EyeMax, Micro-Tech; 6.5-Fr outer diameter, 220-cm working length, and 0.75-mm working channel) was introduced through a short double-balloon enteroscope (EI-580BT, Fujifilm; 3.2-mm working channel), enabling the direct visualization of the stones ( Fig. 1 ), holmium laser lithotripsy, and complete bile duct clearance ( Video 1 ).

**Fig. 1:**
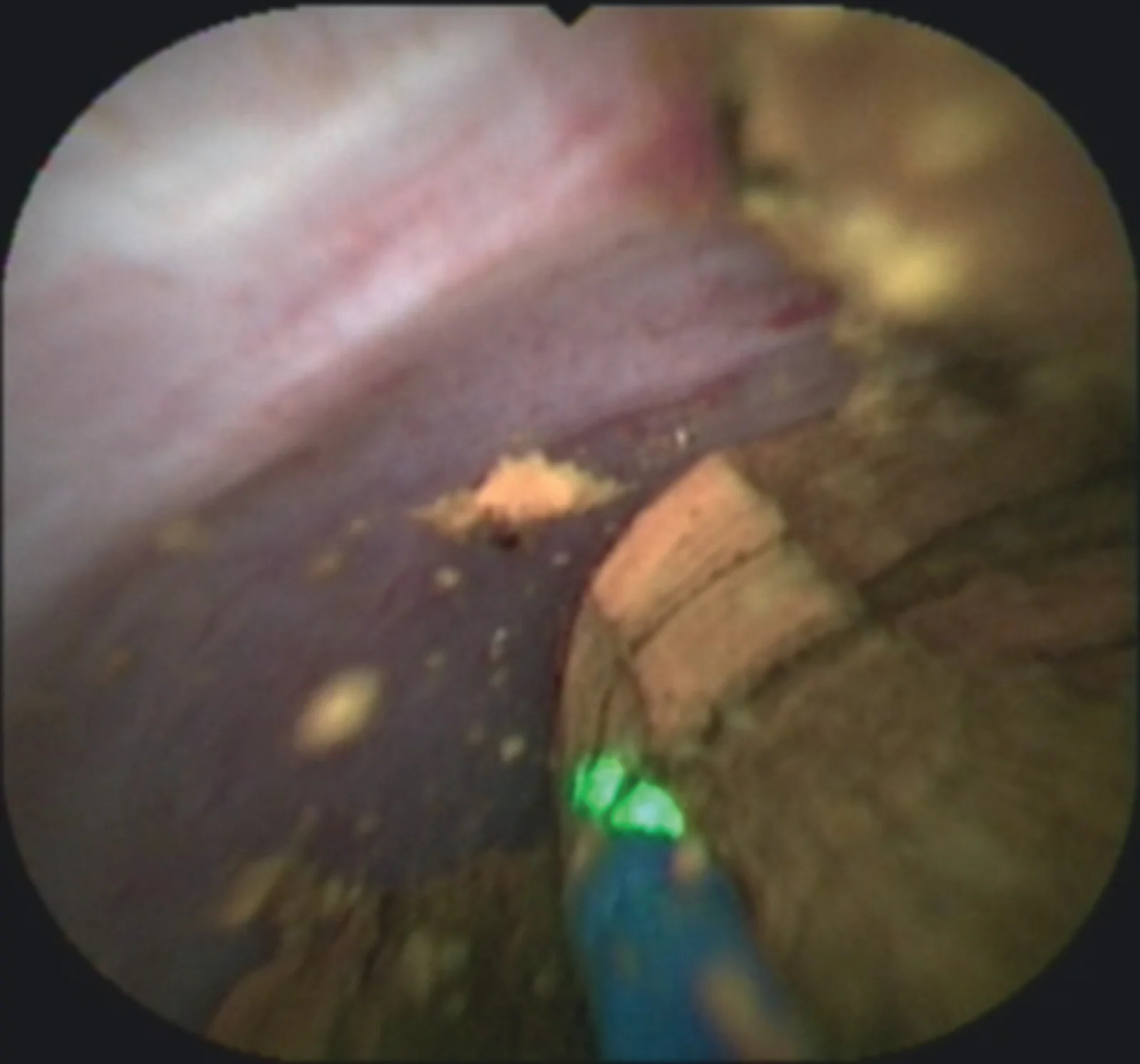
A cholangioscopic view during holmium laser lithotripsy of a common bile duct stone using a slim digital cholangioscope.

**Video 1** Cholangioscopy-guided holmium laser lithotripsy during double-balloon enteroscopy–assisted ERCP using a slim digital cholangioscope in a patient with Roux-en-Y anatomy.


This case demonstrates how the combination of a short double-balloon enteroscope and a slim digital cholangioscope expands the therapeutic capabilities of DBE-ERCP beyond conventional stone extraction. To our knowledge, this is the first reported use of a compatible 6.5 Fr slim digital cholangioscope for holmium laser lithotripsy during DBE-assisted ERCP in a patient with Roux-en-Y anatomy, highlighting a novel minimally invasive treatment option for difficult biliary stones in surgically altered anatomy.

Endoscopy_UCTN_Code_TTT_1AR_2AH

## Declaration of GenAI Use

The authors declare that no generative artificial intelligence was used in the preparation of this manuscript.

## References

[1] Tanisaka Y Ryozawa S Mizuide M Fujita A Watanabe R Hamamura R Peroral cholangioscopy-guided laser lithotripsy using a thin cholangioscope under balloon enteroscopy for Roux-en-Y anastomosis Endoscopy 2025 57Suppl 1E1315 E1317 10.1055/a-2738-7298 PMC12629838 41260579

